# Transference Number Determination in Poor-Dissociated Low Dielectric Constant Lithium and Protonic Electrolytes

**DOI:** 10.3390/polym13060895

**Published:** 2021-03-14

**Authors:** Maciej Siekierski, Marcin Bukat, Marcin Ciosek, Michał Piszcz, Maja Mroczkowska-Szerszeń

**Affiliations:** 1 Faculty of Chemistry, Inorganic Chemistry and Solid State Technology Division, Warsaw University of Technology, Noakowskiego 3 Str., 00-664 Warsaw, Poland; marcin.bukat@gmail.com (M.B.); 01@data.pl (M.C.); mpiszcz@ch.pw.edu.pl (M.P.); 2 Oil and Gas Institute-National Research Institute, Lubicz 25a Str., 31-503 Cracow, Poland

**Keywords:** cationic conductivity, protonic conductivity, polymeric electrolytes, composite electrolytes, ceramic fillers, supramolecular anion traps, transference numbers

## Abstract

Whereas the major potential of the development of lithium-based cells is commonly attributed to the use of solid polymer electrolytes (SPE) to replace liquid ones, the possibilities of the improvement of the applicability of the fuel cell is often attributed to the novel electrolytic materials belonging to various structural families. In both cases, the transport properties of the electrolytes significantly affect the operational parameters of the galvanic and fuel cells incorporating them. Amongst them, the transference number (TN) of the electrochemically active species (usually cations) is, on the one hand, one of the most significant descriptors of the resulting cell operational efficiency while on the other, despite many years of investigation, it remains the worst definable and determinable material parameter. The paper delivers not only an extensive review of the development of the TN determination methodology but as well tries to show the physicochemical nature of the discrepancies observed between the values determined using various approaches for the same systems of interest. The provided critical review is supported by some original experimental data gathered for composite polymeric systems incorporating both inorganic and organic dispersed phases. It as well explains the physical sense of the negative transference number values resulting from some more elaborated approaches for highly associated systems.

## 1. Introduction

The modern world exists in a constant search for energy. The global energy demand is monotonically growing in time. Those facts together with the growing demand for portable devices and increased expectations concerning the sources powering them lead to a situation in which the volume of the worldwide primary and secondary battery market is permanently increasing. Independently EV (electric vehicles) market or stationary power banks have been rapidly developed in the last decades showing the tremendous importance of batteries. Amongst them, lithium metal and lithium-ion batteries (with anode consisting of lithium intercalated carbon, other layered materials, or metallic) are one of the most promising ones, due to their very high discharge capacity and high open-circuit voltage (OCV) (up to over 4 V) [1]. The potential for their development is commonly attributed to the use of solid polymer electrolytes (SPE) to replace liquid ones. The main advantages achieved by the implementation of the said are non-leaking, less flammable electrolyte, higher energy density (as the use of the lithium metal as a negative electrode material is in this case possible), and lower cost due to cell construction simplification. The commercially available liquid electrolytes (as well as the pre-commercialized polymeric designs) are currently based on binary salts, thus, both anions and cations are mobile in them. The typically declared value of the often arbitrary defined “cationic transference number” (t_+_) (more or rather less properly attributed to the cationic fraction of current) is not higher than 0.3. The limited cationic transport in the applied electrolytes is not only related to the inferior operational parameters of the resulting cells but is also responsible for their premature deterioration, especially this occurring at high charge and discharge rates.

On the other hand, the majority of the practically considered protonically conductive polymeric systems such as perfluorinated polyacids [2] polybenzimidazoles (PBI) [3], sulfonated poly(ether ketone)s (sPEEK) [4], polysulphones [5], poly(phenylene)s (sPP) [6], or poly(phenyleneoxides)s (sPPO) [7], along with various polymeric mixtures including blends of non-miscible polymers [8,9] and copolymers [10] or polymer-inorganic filler composites (the materials forming the latter group may be based on Nafion™ [11], PBI [12], sPEEK [13,14,15], sPS [16], or sPPO [17]) are designed as polyelectrolyte type structures, and therefore, they are usually assumed to be sole cationic (protonic) conductors. Consequently, the experiments devoted to the determination of their protonic transference numbers play only a limited role in the investigation of their transport properties. The opposite situation can be observed in protonically conductive systems based either on complexes of polymeric backbones with ammonium salts which, despite their practical applicability are studied for at least thirty years [18,19], or inorganic protonically conductive glasses based on poly (phosphoric acid) [20,21] where both protons and anions can be mobile, and therefore, determination of the protonic transference number value is valuable. Unfortunately, the methodology of such determination is not only significantly less developed in comparison with the lithium systems but, as well, inherently limited by the lack of proton reversible electrodes characterized with stable enough values of their potentials.

The issues related to the ionic equilibria present in the solid polymeric electrolytes were widely discussed by various research teams starting from the early age of the investigations devoted to these materials i.e., an investigation of the conducting species present in polymer electrolytes delivered by MacCallum et al. [2]. Armand concentrated his interest on the charge transfer phenomena occurring at the solid polymeric electrolyte electrode interfaces [22] while Bruce et al. discussed issues related to the DC polarization of the electrolyte itself [23] and transport in associated polymer electrolytes [24]. In consequence of these deliberations both the influence of the interfacial polarization on the determination of the transference numbers [25], as well as, the importance of the enhancement of the cationic transport in the electrolyte (i.e., Jorne [26], Strauss et al. [27], or Arof et al. [28]) were widely discussed.

On the other hand, studying the literature one can find numerous papers claiming either single ion conductivity of the studied systems [10,11,12,13,14,15,16,17,18,19,20,21,22,23,24] or enhanced lithium-ion transport [29,30]. Moreover, in some publications, the transport properties of the polymeric electrolytes are addressed without the meaningful determination of the basic and most prominent charge transport characterizing parameter—cationic transference number. Such an approach was presented for various groups of the materials studied including plasticized [31], hybrid [32], supramolecular compounds based [33] sodium conducting [34,35,36], or even silver ion-based [37] electrolytes. Therefore, it is important to stress that even if the suggested chemical structure of the electrolyte assumes this kind of features, one cannot neglect the counter effect of side reactions occurring in the synthetic step, as well as, the exploitation phase structure deterioration processes causing the existence of the mobile anionic charge carriers. Whereas one can easily find publications neglecting the importance of this factor for the battery performance [38] the majority of authors correlate these two phenomena upon both theoretical analysis performed in a system [39,40] and molecular scale [41,42,43,44,45] as well as, the results of the experimental investigations [46,47].

Despite the very early theoretical consideration originating from near a century ago [48], the former approach has been investigated for about the last thirty years as it is presented by Doyle et al. in their early work [40]. The main problem addressed here is focused on the numerical simulations of the Li|electrolyte|MnO_2_ primary cell performed for various discharge rates and for two different electrolytes—one characterized with t_+_ = 0.2 (typical for (PEO)_x_LiCF_3_SO_3_ systems) and the other being the hypothetic representative of the cationic single-ion conductors (t_+_ = 1.0). Both the discharge curves and concentration profiles calculated here prove that the discharge rate of the cell incorporating the typical polymeric electrolyte is limited by the local depletion of the salt present in the electrolyte within the porous cathode. Contrary to this situation, it does not occur when a single ion conductor is taken into consideration. While it is obvious that anion immobilization leads to a decrease in conductivity. It is, as well, determined that this phenomenon does not inflict the relevant cell performance only if it is occurring up to a certain discharge rate. On the other hand, above a certain load threshold, a decrease of the overall conductivity leads to the deterioration of cell operational properties even if a single cationic conductivity is achieved. Moreover, this work addresses the issue that the assumption of the complete dissociation of the dissolved salt originating from the theory of the dilute solutions cannot be applied towards systems of these kinds and more rigorous concentration solution theory. The based approach (taking into consideration the presence of neutral ionic pairs, as well as, higher charged and neutral ionic aggregates) must be considered to properly identify the correlations between the cationic transference number and the diffusion coefficients. Consequently, the typical methods of lithium transference numbers determination yielding in results ranging from 0 to 1 are of lesser reliability as properly defined and measured transference numbers for cations can be here lower than 0. It results from the presence of numerous neutral and charged species in the solution different from pristine cations and anions. The curious, at the first glance, attribution of the negative values of the transference numbers to the physicochemical phenomena occurring in the electrolyte will be delivered later.

Independently on the discrepancies mentioned above and doubts understanding the transport phenomena occurring in the electrolyte is, surely, crucial for the proper assessing the operational features of the galvanic cell incorporating it. Moreover, the idea standing behind the determination of the lithium (or more general cationic) transference number is relatively obvious. Both the cathodic and anodic reactions of the lithium-based cell involve only cations while anions are either responsible for cell polarization or in less favorable conditions are responsible for the occurrence of the parasitic reactions leading to the premature wear of the electrode materials. Therefore the parameter denoting the fraction of the overall cell current related to the mobility of cations, however, defined, remains an important parameter describing the practical applicability of a given electrolyte in such a system [46,47].

## 2. Materials and Methods

### 2.1. Methodological Background

The analysis of various available methods of the determination of the transference numbers in terms of the problem studied must be performed in two independent categories. The first is related to their general applicability to the solid or quasi-solid highly viscous systems while the other must address the issue of their relevance to this kind of system. A synergetic effect of the low dielectric constant of the polymeric solvent used and the high concentration of the salt added leads to [44] highly associated systems exhibiting significant discrepancies from the idealized theory of diluted fully dissociated solutions standing in behind of the majority of the methods of the determination of the transference numbers. Another early work of Bruce and Vincent [49] analyzes the latter issue dividing described methods into four main classes.

#### 2.1.1. DC and AC Conductance-Based Measurements

The first of them is based either on DC conductance measurements and/or on the application of small-signal AC perturbations all of them assuming that for low fields transport of each charged species is directly proportional to the strength of the field and the concentration. These methods assume that neutral species if, at all exist, remain immobile and do not contribute to the overall current passing through the system. The mentioned assumption is, unfortunately, not valid for numerous systems of interest where an ionic pair’s mobility indirectly caused by the electric field applied occurs. Consequently, the practical interest in these methods is significantly reduced. Sorensen and Jacobsen [50,51] developed an approach in which transference numbers are determined based on the AC impedance experiment conducted in the symmetrical non-blocking electrode-based cell.

Therefore, according to the said authors if the measurement is spanned down to appropriately low frequencies (typically located in the μHz range) the amount of charge transferred in each half cycle of the applied perturbation is sufficient to produce gradients of concentration of the charged species within the electrolyte. Moreover, if the frequency is even more reduced the concentration waves and/or quasi-steady steady-state profiles of concentration may develop. Therefore, it is possible to determine the transference number of the electrode active species upon the values of the bulk resistivity of the electrolyte and its diffusion impedance at a frequency approaching zero. For these types of measurements, one must take into consideration their three main limitations. The first of them, being inherent to the method, is related to its assumptions based on the applied binary model of the non-associated salt solution. The two others are of a technical nature and are related to the instability of the long-lasting measurements (often up to 24 hours). These can be related to either change of the SEI properties during the course of the experiments or the non-stationary nature of the system caused by i.e., mechanical vibrations or convection mixing occurring within the electrolyte layer.

Moreover, the impedance spectroscopy derived methods were used to estimate the ionic and electronic conductivity (and in consequence ionic and electronic transference numbers in multi-component electrode mixture layers of the lithium-ion batteries [52]. A deviation in the type of the transmission line based model element representing the low-frequency diffusion element of the relevant equivalent circuit allowed to distinguish the situations in which the electronic resistance is present and absent in the composite material investigated while its quantitative characterization of the appropriate circuits was a measure of the relevant fractions of charge transport. The detailed analysis allowed, as well, for the determination if the percolation occurring in the material if ionic-electronic or electronic-electronic character. On the other hand, impedance spectroscopy was, as well, applied to determine the ionic and electronic components of the conductivity for the in situ formed composite comprising of the chitosan polymeric matrix, salt, and the copper [53] or silver [54] metallic filler formed upon the chemical reduction of the appropriate salt by the polymer. In this case, deviations of the high-frequency dielectric dispersion observed in the impedance spectrum registered with the use of the stainless steel ion blocking electrodes setup were attributed to the changes in the ratio between the ionic and electronic conductivities of the material studied. Unfortunately, the reliability of the proposed approach has not been carefully validated. Moreover, it must be stressed that methods belonging to this class were found to be of only limited applicability to the polymeric systems. They, on the other hand, were mostly used in a very early stage of the development of solid polymeric electrolytes, and therefore, the number of publications devoted to their use is significantly lower in comparison with other alternative approaches.

#### 2.1.2. EMF-Based Studies—Hittorf Type Experiments

Therefore, in contrast to the class mentioned above characterized with the limited applicability towards solid polymeric systems the second class of electrochemical methods can be distinguished for concentrated electrolytes. Moving boundary [55,56,57] or Hittorf and Tubandt type experiments (please refer to [58,59,60,61,62] or a view on very specific modifications/applications of this 19th century originating experimental technique), as well as, techniques based on measurements of the EMF performed on the appropriate concentration cells or even quite exotic measurements performed in force fields i.e., in the case when the measured sample is placed radially on a spinning motor. From the general experimental point of view, only the E.M.F.-based experiments revealed their applicability potential towards the solid polymeric systems. On the other hand, there are some specific situations in which Hittorf type measurements can be applied i.e., Leveque et al. [63] applied it to a very specific highly cross-linked material for which a sandwich-type cell can be assembled from numerous non-adherent slices of the studied material. In this particular case, it was used only to prove the single ion transport in the material in which the anion was chemically bound to the polymeric backbone. Other highly specific situations were described in the works of Olsen et al. [64] where a hybrid polymeric electrolyte was investigated or Vassal et al. [65] where a modified Hittorf method was used for a polymeric oxyethylene based membrane doped with KOH and plasticized with the addition of water. A very low cationic transference number equal to 0.07 was determined for potassium in a fully amorphous matrix while the reference sample revealed t_+_ = 0.28. Moreover, Vincent [66] reports the applicability of such a method to study the cation mobilities of PEO Hg(ClO_4_)_2_ and Mg(ClO_4_)_2_ electrolytes. It was found that contrary to the Mg^2+^ cation, which is totally immobile, the mercurium Hg^+2^ one reveals significant mobility in the polymeric host.

Finally, Bruce et al. [67] investigated the possibility of the application of the described method to a melted polymer electrolyte working in 120 °C. An in-house built, modular, four-compartment cell was used to separate the catholyte, anolyte, and two reference areas. An interesting idea based on the utilization of lead, and not lithium cathode, allowed us to determine the quantity of the lithium deposited on it. By this means, the overall charge applied to the cell could be divided into two parts: one related to the reaction of interest (lithium plating/stripping) and the other attributed to various parasitic reactions (e.g., polymer host decomposition) occurring in the cell. Results of four independent experiments revealed values of t_-_ in the range of 0.91 to 0.97 resulting in an average of 0.94 ± 0.05. This value is surprisingly higher (or otherwise the t_+_ is significantly lower) than the same parameters determined by other methods. The observed discrepancy can be here obviously attributed to the inherent incompatibility of the method with the non-ideal associated systems. In addition to that, the combination of the said approach with the theoretical considerations was presented in [68] where it was applied to m-PBI membranes doped with the phosphoric acid operating in elevated temperatures. Whereas no direct determination of t^+^ was described here the diffusivity of the phosphoric acid moieties, and therefore, anions were determined as a function of the water vapor pressure for two different operational temperatures of the material.

#### 2.1.3. Concentration Cells Based Experiments

On the other hand, the most recognized method from this class is, based on the assumption that the electromotive force of the M|POLMX_c1_||POLMX_c2_|M (where POLMX_ci_ stands for complexes of a given polymeric host POL with salt MX of concentration ci) cell depends on the activities, not concentrations which are variable with the concentration of the salt (or other words to the polymer to salt ratio). It is impossible to determine the transference number without determining the combined activity of the ionic species in the polymeric solution from either the independent electrochemical experiment [69,70] or using the addition of an additional redox pair into the electrochemical system under investigation [71]. An even more elaborate set of concentration cells based experiments leading to the determination of the transference number for a highly concentrated polymeric electrolyte as described in [72]. Both cation reversible Li electrodes and anion reversible Pb|PbI_2_ ones were here combined in four different combinations of the concentration cells. Two of which are allowed while two others are disallowed for the ion’s migration between the cell compartments. This allows for omitting the need to determine the activities of the ionic species present in the system. A high, and in some cases higher than 1.0 value of the cationic transfer number, determined by these experiments was on the one hand attributed by the authors to the presence of positively charged ionic aggregates but on the other, this conclusion is “softened” by the observation that the values exceed unity only within the range of their possible experimental error. The EMF measurement-based method was applied to mercury ions transporting Nafion membranes [73] and PVC base membranes [74]. Moreover, it is worth stressing that the modification of this method can be, as well, applied to distinguish between the ionic and electronic constituents of the conductivity in the mixed conductors utilized in the fuel cells [75]. Moreover, it was, as well, applied to Ba(Zr_0.8_4Y_0.15_Cu_0.01_)O_3−δ_ type systems [76] where a portion concentration cell was formed while two opposite sides at the analyzed specimen were faced against water vapor of different pressures. A further modification of the classical EMF measurement-based approach to protonic systems was delivered in [77] where the Gorelov (active-load) method of electrode-polarization correction was applied by introducing a variable resistor into the experimental setup placed in parallel with the investigated cell. It must be, anyhow, stressed that the physicochemical rationale standing behind this particular application is significantly different than the above described one. On the other hand, a comparison of the results of the Hittorf method and E.M.F. based determination of the counter-ion transference numbers performed in ion-exchange membranes was delivered in [78].

#### 2.1.4. Non-Electrochemical Measurements—Diffusion of Radiotracers

The third class of the experiments described in [49] comprises all of these with some exemption to Cottrell Equation-based experiments [79]; non-electrochemical methods which are prone to the diffusion originating flux comprising of all not only charged species. Here one can also find a solid-state chemistry classic–radiotracer diffusion method and pulsed-field gradient NMR-based investigations. The former were tested against their applicability towards polymeric ionic systems by Chadwick et al. [80,81,82] for the PPO-NaSCN system were ^22^Na and ^14^C isotopes laced salt can be used as a tracer source. It is considered here that the drift velocity of ions affected by the force of the unitary strength is independent of the nature of the force applied. Therefore, the electromobility of ions and their diffusion coefficient can be related to each other by means of, for example, the Nernst–Einstein equation. Here we meet the main limitation of the method—the equation mentioned above is valid only for an ideal non-associated solution, which is not the case for the systems of practical interest. In this case, an introduction of the conductivity mechanism dependent and the salt concentration correlation coefficient is needed. Moreover, applying this method to semi-liquid systems, one meets severe technical complications related to a proper determination of the radioactivity profile within the sample under investigation. In addition to all drawbacks mentioned above of this method, one must, obviously, notice its absolute incompatibility with the lithium-based systems as the two most known radioactive isotopes of lithium ^8^Li and ^9^Li reveal t^1/2^ equal to 838 ms and 178 ms, respectively. All these make this method rather an exotic curiosity in this field of interest, not a valuable experimental tool.

On the other hand, the radiotracer method originating results were contrasted with the ones originating from the conductivity-based diffusion studies for PEO_x_NaI systems (x = 52.20) by Fateux et al. [83]. The measurements were performed in a temperature range above 65 °C, hence, the sample can be treated as a mono-phase amorphous visco-liquid material. It was found that the cation diffusivity coefficient value is about half an order magnitude lower than the one characterizing the anion in all the studied temperature range reaching 400 K. For the more diluted sample conductivity data-based salt diffusion coefficient (determined by means of the Nernst–Einstein equation and, therefore, bearing all the limitations related to it) is for the whole temperature range studied almost identical with the radiotracer determined anionic one. For the more concentrated sample, the diffusivity of salt is a few times higher at 340 K with the difference almost completely vanishing at the highest temperature studied. The difference between the behavior of these two samples can be interpreted in terms of the weak (x = 52) and strong (x = 20) impact of the ionic associations on the ionic transport properties. Finally, the t_-_ value is determined based on the diffusion coefficients (ignoring the mentioned effect) to be equal to 0.75. Another system with mobile sodium ions being the colloidal ion exchange membrane was tested by the same method by Brady and Salley [84]. Both ^22^Na and ^24^Na isotopes were here used as tracers, in the case of the latter, either added as an external constituent or obtained in situ by the neutron irradiation of the ^23^NaCl-containing material.

#### 2.1.5. Non-Electrochemical Measurements—PFG NMR Experiments

The other nucleus-oriented method is based on the NMR investigation of the diffusion coefficients of the species containing NMR-prone nuclei. In the typical case of polymeric electrolytic systems, these are ^7^Li and ^19^F belonging to numerous anionic species. Once again a strong discrepancy between the self-diffusion coefficients of ions D_+_ and D_-_ corresponding to the cationic and anionic transference numbers and the values measured by the PFG (Pulse Field Gradient) experiment which, as well, include a contribution from all other species containing appropriate nuclei including the neutral ion pairs/aggregates and charged ionic multiplets. The principle of the measurement for the polymer electrolyte was established by Berthier et al. [85] and Whitmore et al. [86,87] for the polyether-LiCF_3_SO_3_ systems. Independently of this disadvantage the PFG NMR-based methods are widely accepted and utilized for the systems of interest i.e., [88] where it was utilized for PvDF-HFP (poly(vinylideno difluoride- hexafluoropropene copolymer) gels [89], systems containing supramolecular anion traps [52,90], other systems with enhanced lithium cationic conductivity [91,92] to water in salt electrolytes [93], and basic studies devoted to the effect of polymer host molecular weight of the polymer host and the salt concentration on the transference numbers in the electrolytes [94]. Moreover, the temperature [95,96,97] and pressure as well as, its electrophoretic modification [89] are widely accepted and utilized for the systems of interest, therefore. A deeper insight into the discrepancies between results originating from it and the values of the t_+_ determined by the electrochemical methods is provided below in the sections describing the more elaborated electrochemical methods of the transference number determination.

#### 2.1.6. DC Polarization Experiments

The fourth class of methods distinguished by Bruce and Vincent according to [49] is based on the measurements of the current passing through the sample after engaging the DC polarization. An example of the comparison of the results gathered by both PFG NMR and the said electrochemical approach is delivered by Shigenobu et al. in [98]. Whereas the presented paper does not address directly polymeric electrolytes the solvated systems presented there reveal numerous similarities to these materials of interest, and therefore, are worth noticing. Similar to the methods mentioned above gathered in group three, they involve the transport of both charged and neutral species. On the other hand, what makes them significantly from those already described is that, contrary to the previous ones, their mobility is undistinguishable here. The presence of both electrical and chemical potential gradient changes how the charged species contribute to the overall net flux in the sample under examination distinguishing their impact on the value of the transference number from the one delivered by the neutral species.

#### 2.1.7. Microelectrodes-Based Experiments

An interesting approach, being different from the “gold standard” created here by Bruce and Vincent’s belonging, as well, to his class was created by Farrington et al. and is based on a simple amperometric measurement performed twice first time with the use of micro and the second with the macro disk electrodes. The theoretical investigations are delivered in [99] claiming that for the microdisk the diffusion length of an electroactive ion becomes much larger than the radius of the electrode in consequence of what the diffusion field becomes hemispherical and reaches a steady state. The opposite assumptions should be made for the macrodisk case where the planar diffusion conditions are achieved with a diffusion length much smaller than the electrode radius. Due to this discrepancy, the current densities for both measurements depend on the salt diffusion coefficient at different power exponents what allows to determine the diffusion coefficient upon their comparison. In the next step, the D (diffusion coefficient) value can be recalculated into t (transference number) what, of course, is biased with the assumption of the solution full dissociation and ideality of the solution investigated what is, in general, far from the situation found in polymeric electrolytes. The more detailed experimental validation of the proposed method is delivered in [100] with the use of 50 μm and 2 mm gold disk electrodes. It is worth stressing that in this interesting experimental setup, a set of blocking electrodes can be applied to the determination of parameter typically achievable only by the application of ion transporting electrodes what leads to a significant simplification of the measurements, especially if, more “exotic” (ionic moieties such as CF_3_SO_3_^-^, TFSi^-^ - (CF_3_SO_2_)_2_N^-^ or FSi^-^ - (FSO_2_)_2_N^-^ for which anion reversible electrodes are virtually unavailable) ions are considered. Another galvanostatic polarization-based approach this time based on the current flux performed in the transporting electrodes setup correlated with the determination of the dendrite growth-related short-circuiting time observed for an electrolyte layer of a known thickness was proposed by Schaefer et al. [101] and tested against high lithium transference numbers exhibiting 3-D networked electrolytes.

#### 2.1.8. Isothermal Transient Ionic Current Type Approach

A completely different methodology based, as well, on the DC polarization technique was proposed by Watanabe et al. [102] for polymeric electrolytes as a modification of an Isothermal Transient Ionic Current (ITIC) approach used previously to determine ionic mobilities in a dielectric material such as SiO_2_ and insulating polymers. Based on the experiences gathered for SiO_2_ where the significantly differencing mobilities of Na^+^ and K^+^ ions were independently determined within one experiment authors suggest here that in the case of the polymeric electrolyte the anion and cation mobilities could be found in the same manner. To verify this assumption a PEO-LiSCN complex was examined at first by means of the impedance spectroscopy to determine its conductivity and later subjected to rapid polarization changes to register the transient currents. In the first step, this experimental approach was applied to the pristine, salt-free polymer host to determine the current fraction originating from the polymer dipoles reorientation, not from the ionic mobility. That fraction was found to be small enough to be neglected in the subsequent experiments.

In consequence of this observation, the salt-containing samples were polarized being sandwiched between either blocking electrodes made of platinum or the non-blocking lithium ones. After a given time (during which a current decrease was observed—which is related to the accumulation of the charge carriers in the vicinity of the electrodes blocking them) the sign of polarization was reversed and the current dependence of time was registered once again. A time at which a current maximum or maxima were observed is here attributed to the release of the accumulated carriers into the bulk of the electrolyte. In the case of Pt electrodes (blocking to both cations and anions) two time separated peaks (related to the separated pools of anions and cations differing in their mobility) are observed while for the Li ones there is only one (as cations can be transported through the electrode-electrolyte interface, and therefore, do not result in a separate maximum) obviously related to the anions mobility. Finally, the time of ion flight is assumed to be reciprocally proportional to the mobility of the corresponding ions (μ_x_ ~1/τ_x_). The transference number of both charged species are on the other hand calculated upon the ideal binary solution assumption according to Equation (1).

(1) $$t_{x}\frac{\;={\;\mu }_{x}}{\left(\mu_{c}+\mu_{a}\right)}$$

for which index _x_ is _c_ or _a_ for cations and anions respectively. The same technique was verified by its inventors as applicable to the studies of the thermal dependencies of the ionic mobilities (and therefore the transference numbers) for a poly(propylene oxide) based networked material doped with LiClO_4_ [103] and with alkali metal thiocyanates [104]. What is quite astonishing is that in all three cases reported here, the polarizations applied were in the range of 2.5–5 V which provides doubts related to the electrochemical stability of the constituents of the materials studied e.g., thiocyanate anion is prone to oxidation, perchlorates are easily reduced to other chlorine-containing anions, as well as the electrochemical stability window of the studied polymers does not exceed 4 V. More examples of the utilization of the described method can be found in [105] where it was utilized in the case of the ionic liquid-ionic glass dispersion, in [106] where it served as a measure of not only migration but, as well, the creation of the Ag^+^ ions in silver iodide and in [107] where the problem of the mixed ionic-electronic conductivity in the glassy electrolyte was investigated. Moreover, polymeric systems with mobile zinc [108] and copper [109] ions, as well as, single-ion conductors [110] were successfully investigated using this and similar methods. On the other hand, it is worth noticing that the above-described issue of the electrochemical stability of the studied material under the polarization originating stress does not cover the inorganic materials studied in the first three of the six papers mentioned latterly.

#### 2.1.9. Wagner’s Method and Its Modifications

Doubts of similar nature are valid for a method introduced by Wagner (please refer to [111] for a list of his numerous contributions) playing a role of a gold standard of the solid-state chemistry (for example, see [112]) in terms of the determination of the ionic and electronic constituents of overall conductivity of the material under investigation, and therefore, the electronic (t_e_) and ionic (t_i_) transference numbers characterizing it. The experiment is based on the polarization of the non-symmetric cell M(s)|material |X(s) (where M(s) represents transporting and X(s) blocking metallic electrodes) with a DC polarization of an amplitude high enough to cause the concentration gradient of the mobile cations (M^n+^) but on the other hand small enough not to cause the electrochemical reaction. The initial and stationary state currents are registered to determine the sum of the ionic and electronic and purely electronic currents, respectively. In addition to that if the measurements are performed at few different polarizing potentials it is possible to distinguish between the electronic and hole constituents of the conductivity. As the latter problem remains usually out of the interest of the polymeric electrolytes devoted to research the experimental setup is often simplified by utilization of two blocking electrodes and the ratio of currents is registered as an only parameter yielding, finally, in t_e_ and t_i_ values only.

Independently of the above-described doubts, the above-described Wagner’s method [113] is from time to time applied to the polymeric electrolytes with both metal ions [114,115], as well as, protonic conductivities [116,117,118,119,120,121,122,123]. Therefore, it is worth analyzing both its proper and misused applications. At least some of these applications suffer relevant deficits i.e., a polymeric electrolyte based on the poly(ethylene oxide)/poly(vinyl alcohol) mixture doped with cobalt chloride (note as a curiosity that CoCl_2_ 6H_2_O hydrate was used as a doping salt that enriched the system’s composition with a significant amount of water) was tested against its electronic conductivity. A silver (blocking) and silver–silver chloride (anion reversible) electrodes were used for the said test with the applied potential difference equal to 1.5 V. Upon the results, the authors claim that the ionic transference number for the system studied is in the range t_i_ = 0.85 while, therefore, the electronic fraction of current is equal to about 15% of the overall current flux. This conclusion is unjustified upon the chemical composition of the sample as there is an absolute lack of moieties in it being able to delocalize electrons, and therefore, transport them along with the sample. The obvious, and completely contrary to the delivered conclusion is that the applied potential exceeds the electrochemical stability window of water present in the sample as a constituent of the doping salt. Therefore, the steady-state current is related not to electronic conductivity evidently absent in the sample but the water electrolysis process.

Similar experimental shortcomings originating from the irrelevant application of the Wagner’s method were, as well, described for the poly(vinyl acetate) NH_4_SCN electrolyte, as well as, for a PAN-NH_4_Cl system and in the case when the polarization potential reaching 1.5 V was applied to PVP-KIO_3_ electrolyte, and to both pristine and glycerol plasticized methylcellulose based protonically conductive systems. Whereas the application of the same method to the PEO-NH_4_I-Al_2_O_3_ [124] when performed carefully (thus with a polarization potential equal to 200 mV what assures lack of the parasitic electrochemical reactions) yields with t_i_ equal to 0.99 confirming lack of the electronic conductivity in the system studied. An example of an improper experimental setup leading to the doubtful results of Wagner’s method can be on the other hand found in [125] where its application is improperly linked with the transient ionic current type measurements. Moreover, Woo et al. [126] proved that Wagner’s technique can be combined with Watanabe’s AC impedance technique [127]. This combination allowed us to determine the value of the total ionic transference number of 0.996 with protons originating constituent equal to 0.21 for the caprolactone-based polymeric system. Another interesting combination of the transference number determination techniques was presented by Hashmi et al. [128] for the PEO-NH_4_ClO_4_ solid system and by Chandara et al. [124] and Mayura et al. [129] for its PEO-NH_4_I analog. In both cases, Wagner’s approach was used to distinguish between ionic and electronic constituents of total conductivity (neglecting, in general, the occurrence of the electron transport phenomena) while A Hittorf approach-based polarization experiments were used to split the overall ionic current into cationic and anionic fractions. Moreover, some authors consider theoretical deliberations depicting the dependence of the proton transport mechanisms on water content applying to them either MD simulations [130] or a condensed media theory-based approach [131]. Even if no direct values of the t^+^ parameter are delivered by these contributions the provided theoretical insight is a valuable addendum to the results of the above-cited experimental works.

Somehow inconclusive research based on the application of the simplified Wagner’s experimental setup to clay-PEO composite is delivered in [132]. Similar experimental errors seem to responsible for the determination of the electronic constituents in the range 0.02 to 0.08 for various potassium ion-based systems [133]. On the other hand, the same method applied allowed Arya et al. [134] to neglect the electronic contribution based on very similar results (t_e_ = 0.01 to 0.05) yielding from the 20 mV polarization of PEO-PVP self-standing films. Similar in nature discrepancies were observed in [135] where an electronic contribution reaching 20% of the total sample current were astonishingly determined for a gel-like LiBF_4_ electrolyte with the application of an unspecified polarization potential. It was also applied to PVC NH_4_CF_3_SO_3_ ionic liquid plasticized electrolyte [136] revealing a surprisingly high electronic constituent of the conductivity (0.18) even though the polarization voltage was reasonably low (0.5 V).

Moreover, the same method can be carefully applied to systems in which electronic conductivity can really occur. It delivers valuable information on the nature of the charge transport processes present in the material studied. A series of the in situ formed composites was formed by Kumar et al. [137] upon the sulphuration of the cooper sulfate performed directly in the polymeric PEO NH_4_ClO_4_ matrix. While the fraction of the nano-dispersed CuS was increasing from 0 to 5 wt% the value of the electronic transference number was increasing from 0 (being an obviously correct value for purely ionically conductive PEO-NH_4_ClO_4_ system) up to 0.3 for the maximal amount of the dispersoid. Wagner’s method with polarization voltage equal to 0.3 V prevents all parasitic electrochemical reactions possible to occur upon polarization. On the other hand, an increase of the overall conductivity of the sample was observed being possibly caused either by the occurrence of the electronic constituent of the conductivity or by the changes in its ionic part related to the activity of the filler similar to the one described in [138].

Another valuable application of Wagner’s method can be found in a paper delivered by the same research group [139] where it was applied towards various composites of the photonically conductive polymeric electrolytes with semiconductors such as PbS, CdS, etc. Application of the relatively small polarization (0.1 to 0.2 V) together with a carefully set experimental setup allowed us to determine that the electronic constituent of the conductivity is in the range of 0.05 to 0.2 depending on the dopant type and amount. A review of technical issues related to the application of this method can be found in [140] whereas its four-electrode probe-type modification is carefully described in [141,142]. It is worth stressing that theoretical issues related to the possibility of the electronic constituent in the conductivity are delivered in [143] based on the first principle computer modeling of the PEO-LiTFSi system.

#### 2.1.10. Coupled Electrochemical Techniques

A combination of the simplified Wagner’s method based on the chronoamperometry measurements realized in the blocking electrodes setup with the impedance ones performed in the sodium reversible amalgam electrodes was used by Mishra et al. [144] for a nanocomposite gel polymer electrolyte. Whereas Wagner’s experiment meaningfully allowed here to neglect the electronic contribution to the overall conductivity the fraction of the charge transported by sodium ions was doubtfully determined using the Sorensen–Jacobsen method based on the impedance measurements results performed down to 1 Hz only instead of the MHz range proposed in the original contributions introducing the method [50,51]. Another combined experiment was described by Perera et al. [116]. Simplified Wagner’s setup with relatively large 1 V DC polarization allowed to find the suspiciously electronic contribution ranging up to 0.15 for a gel-type electrolyte. On the other hand, the polarization experiment performed in an iodide reversible electrodes setup allowed to determine the anionic transference number equal to 0.79. A combination of Wager’s approach and Transient Ionic Current experiments was applied to a protonic polymeric conductor based on a PVAc-NH_4_SCN [145]. The results were here gathered with the use of the polarization potentials equal to 0.85 V and 2.0 V, respectively. As these are relatively high and at least in the latter case can easily exceed the electrochemical stability window of the system studied both an electronic contribution equal up to 0.06 and relatively high determined ionic mobilities can be biased with the impact of the parasitic electrochemical reactions on the image observed.

A combination of complex impedance measurements performed in the lithium electrodes symmetrical setup with various amplitudes of the AC perturbation ranging from 5 mV to 1.1 V with the polarization experiments performed in potentiostatic regime with the potential values ranging from 5 mV to 3 V was applied by Watanabe et al. [127]. Three kinds of in-house synthesized amorphous networked polymer electrolytes were investigated. A special experimental procedure in which the polarization experiments were carried out with increasing a potential stepwise was introduced. An intermediate step allowing for the equilibration of gradients present in the material was added, and therefore, after one polarization experiment, both terminals of the cell were short-circuited to achieve the depolarization. After confirming the depolarization by an electrometer, the next experiment was carried out. From the impedance spectra registered the values R_b_ (bulk resistance of the material) and R_e_ (charge transferee resistance occurring at the electrode-electrolyte interface) were separated corresponding to the high and low-frequency semi-arcs, respectively. Polarization experiments were held until a steady-state current was achieved. Finally, the cationic transference number was determined according to Equation (2):

(2) $$t_{+}=\frac{R_{b}}{∆{V/I}_{s}-R_{e}}$$

#### 2.1.11. Polarization Method

Therefore, despite the relative experimental simplicity of all the approaches mentioned above, the most popular solution belonging to this class was created by Bruce and Vincent and their coworkers and is described in detail in their earlier papers [146,147]. The original idea of the experiment is based on the polarization of the electrolyte sample placed between cation non-blocking electrodes (M(s)|MX-pol(s)|M(s)) with a small (10–50 mV) constant potential difference. The initial current (Io) observed in the polarization experiment is, thus, determined only by the conductivity of the electrolyte. After a certain time, the charge transport is limited only to the species being reversible against the electrodes (cations) and the current falls to a steady-state value Is. This phenomenon is related to the formation of a linear salt concentration gradient within the sample. If small values of the polarization potential are considered a linear dependence between the perturbation and the steady-state response should be observed. Therefore, if one considers the relevance if the Nernst–Einstein equation to the system studied the t+ value can be here simply defined as a ratio Is/Io. This oversimplified approach was introduced by the same authors in [148] and initially tested for a linear poly[(alkoxy)phosphazene], [NP(OC_2_H_4_OC_2_H_4_OCH_3_)2]_n_ (MEEP) complex with lithium salt (LiSO_3_CF_3_)0.25·MEEP [149]. It was also applied to the single ion conducting polymer-silicate nanocomposites by Kurian et al. [150] and to poly[lithium tetrakis(ethyleneboryl)borate] based SICs by Cakmak et al. [151].

That approach is burdened with both technical and inherent discrepancies. While the latter ones as specific to the method are described in numerous publications (i.e., [152] originating from the same research group) the first group of them can be at least partially overcome and, therefore, requires corrections for finite electrode kinetics and/or the development of the electrode passivation. The most popular one is related to the application of the electrochemical impedance test prior to and after the polarization to estimate the changes in the resistivity of the passivation layer, and therefore, to eliminate the current changes related to it form the overall image of the sample under investigation. The values of solid electrolyte interface (SEI) resistance before (R_0_) and after (Rss) dc-polarization are in consequence incorporated into the Equation (3) used for the t+ value calculations:

(3) $$t_{+}=\frac{I_{s}(∆{V-I}_{0}R_{0})}{I_{0}(∆{V-I}_{s}R_{s})}$$

Yielding finally in a widely recognized equation named after its authors; the Bruce–Vincent formula.

Moreover, the authors claim in [49] that it is impossible to derive transport numbers directly from this kind of experiment if mobile neutral ionic pairs are present in the studied material. This inherent drawback of the technique proposed does not diminish the practical importance of the measurement of this kind as the combination of the chemical and electrical gradient is encountered regularly in practical devices such as batteries under load. Therefore, a more detailed analysis is performed to define the limitations of the method originating from ionic associations observed in virtually all systems of practical interest. Considering the concentration gradients of cations and anions developed under the electrical polarization one should keep in mind that ion pairs forming is a dynamical process, and therefore, the concentration gradient of ion pairs (not being directly mobile in the electric field as the neutral species) is created as well. Consequently, the net flux of anions due to their electromobility is balanced by the diffusive counter-flux of both anions and ion pairs. This leads to the situation in which the diffusion coefficient of the neutrals species (ion pairs) significantly inflicts the steady-state effective conductivity of the polarized sample, and thus, the I_s_ value.

Depending on the ion associations present in the sample two boundary situations must be considered one in which the concentration and/or the diffusivity of the ion pairs are small in comparison with those of anions and the second when the ion pairs dominate in the system. In the former case, the steady-state current is determined by the mobility of cations and proportional to the value of D_+_ only while in the latter the anion flux is almost fully balanced by the flux of ion pairs, and that current is proportional to the sum of D_+_ + D_−_. The details of it were described in a paper delivered by Cameron et al. [153]. Upon theoretical divagations a quite astonishing conclusion claiming that a lithium battery could operate successfully even if t_+_ = 0 is drawn becoming fully understandable if one considers the real meaning of the t_+_ value and assume that there is a plentiful supply of mobile ion pairs in solution.

Despite the importance of the ion pairs for the battery operation confirmed as well in [49] some more conclusions can be drawn here. It must be, therefore, stated that unlike the case of the fully dissociated salt solution, the I_s_/I_o_ ratio (for a constant potential possible to be depicted, as well as, the σ_eff_/σ ratio) does not represent the transference number of cations as important ion pairs originating from the fraction of the I_s_ current are present. Moreover, it does not even deliver the information on the average transport parameter of all species involved in the current flux. On the other hand, trends of such measured values if observed with varying temperature, composition, etc. yield valuable insights into the nature of the transport phenomena present in the studied material. It must be also pointed out that the presence of charged aggregates such as triplets further complicates the situation.

Therefore, independently of the specific features mentioned above of the Bruce–Vincent method it can be clearly assumed to be a workhorse of the transference number determination in the polymeric electrolytes. Its application spans each solid polymer electrolyte (but not limited to solid polymers by different authors) like systems of a very elaborated architecture such as materials incorporating metal-organic frameworks [154], hyperbranched hybrid inorganic-organic systems [155], or materials incorporating boron-based [156] or supramolecular anion traps applied a sole modification [52,157,158,159] or in a combination with an inorganic filler [46]. The role of the inorganic filler itself as a cation conductivity promotor was studied as well by the same research group [160]. Copolymer electrolytes [161,162,163,164,165], a crosslinked system based on methoxy compounds [155,166,167,168], plasticized electrolytes or polymers enhanced by mesoporous ceramic fillers [45,61] were successfully analyzed by Bruce–Vincent method. Reasonable values of transference number were obtained as well for quasi liquid systems like ionic liquids [169,170,171] or much more complex systems with porous polymeric membrane enhancing cationic conductivity [172,173,174]. Bruce–Vincent method is as well successfully applied in single ion-conducting polymer electrolytes [175,176,177,178,179] The simplicity of the method makes it most widely used amongst all. The most important conclusion concerning the Bruce and Vincent method is that reasonable results are achievable only if the method is applied carefully and properly.

#### 2.1.12. Concentrated Solutions Theory Approach—Newman’s Method

A detailed study of the divergences introduced to the course of the polarization experiment by the electrolyte non-ideality is delivered by Doyle and Newman in their concept paper [180]. The equation relating the potential gradient occurring due to both current flow and the concentration variations is here proposed in a form that takes into consideration the molar activity confidence of the salt. In the subsequent step basing on the assumption that ionic conductivity, transference number, as well as, the thermodynamic factor can be treated as constant the governing equation is numerically solved for both the initial and the stationary state of the cell considered. Finally, a modified equation for I_s_/I_o_ is proposed as an extrapolation of Bruce–Vincent’s formula (Equation (2)) towards the non-ideal associated systems. Contrary to the original situation the value of t_+_ determined by means of the extended formula does not have to be limited into the range from 0 to 1. The meaning of such can be attributed to the transport of ionic aggregates and will be discussed in the later sections of this paper. On the other hand incorporating the ionic conductivity, salt diffusion coefficient, and the thermodynamic factor into the equation allowing for the determination of t_+_ means that without knowledge of these factors it is impossible to determine it.

Moreover, it clearly stated that very large errors in the values of the transference number determined upon ignoring the differences between the results yielding from the polarization method with the Bruce–Vincent correction and the approach proposed by Newman and coworkers. As an example a concentrated (2.58 M) NaCF_3_SO_3_ solution in melted poly(ethylene oxide) (t = 85 °C) is characterized with “real” t_+_ = −4.38 (!!) while the I_s_/I_o_ value from the Bruce–Vincent experiment returns the positive number equal to 0.37. It is, as well, stressed that the latter number cannot be interpreted as sodium ion transference number while, on the other hand, it delivers a useful piece of information about the transport properties of the material investigated. Moreover, this observation is in agreement with the conclusions of the previously cited contribution of Bruce and Vincent [49]. The authors claim, as well, that is not true that the limiting current fraction is strictly relevant to the practical performance of the electrochemical devices. This observation is due to the significant development of the concentration gradients with the current density increase especially significant while the high current density operation of the cell is deliberated what confirms the theoretical studies delivered by the same team in [181]. It is also noticed that the value of the transference number is related to the magnitude of the concentration gradients which are developed within the electrolyte layer, while the corrections related to the value of the activity coefficient determine how the resulting cell potential is affected by these concentration gradients. Authors conclude that the concentration cell originating data delivering information about the activity coefficients can be combined with the results gathered by other methods to isolate the “real” transference number values. One should note, as well, that to do so in each case the value of the salt diffusion coefficient determined by the independent experiment is still required. From three considered methods (galvanostatic, steady-state, and impedance) authors suggest the former as the most promising one in terms of the establishing of the complete method of the ‘real’ transference number determination.

Therefore, an extended electrochemical model was proposed by the research group headed by Newman [180] allowing the proper determination of the transference numbers without assuming that the solution under investigation is either ideal or dilute. For this purpose, a macroscopic model of the system was employed with three independent species considered. These are cation, anion, and the polymer host. This assumption does not mean that the presence of any other species (pairs, ionic triplets, and higher charged, as well as, neutral aggregates) is neglected. From the thermodynamic point of view, only three independent species exist, and if the system is considered without regard for microscopic speciation the values of the concentrations of all other species existing within it are strictly determined by these three through the fast, and therefore, reaching their equilibria reactions of ion exchange and aggregation.

In consequence, three independent physicochemical parameters are needed to fully describe the state of the system studied. For the method described this set consists of material-specific conductivity (κ), salt diffusion coefficient (D), and the cation transference number (t_+_). Moreover, the pairwise interaction parameters should be introduced (D_i,j_) (where i, j are: + for cations—for anions and 0 for the polymer host).

While the conductivity and the diffusion coefficient can be easily determined by means of the AC. impedance and restricted diffusion experiments respectively (see the details in the experimental section) the measurement of the transference number is complicated by the solution’s non-ideality. Therefore, the determination of the so-called thermodynamic factor from an independent electrochemical experiment is needed to correctly determine the value of the parameter of interest. Upon the set of equations described in detail in [182], it can be found that the potential of the concentration cell M(s)|MX(c_1_)|MX(c_2_)|M(s) can be applied to determine the t_+_ value for cation M^+^. Moreover, neither the reliable method of determining that for the polymer electrolytes not based on the previous determination of t_+_ has not been developed nor the classical methods of the transference number determination are here useless as it is extremely difficult to find the electrode reversible against the typical anions applied in the polymer electrolyte research such as CF_3_SO_3_^-^ or TDI^-^ [183].

Therefore, a dedicated set of two similar but orthogonal experiments is proposed by Newman et al. [180] to determine the values of both the transference number and the activity coefficient as a function of the salt concentration. First of them is a “standard” concentration cell experimental setup in which the salt concentration (or particularly the EO:MX ratio) in one of the half cells is fixed (m) while in the other varies (n). The resulting set of cell voltages (U_m,n_) is gathered as a function of the variable concentration and further combined with the results of the second experiment basing on the short time galvanostatic (constant current) polarization of an M(s)|SOL:MX(c_x_)|M(s) cell (c_x_ corresponds here to various values of the n ratio). The timespan is tuned in a manner allowing the buildup of the concentration gradient in the cell without its propagation into the center of the electrolyte layer. Therefore, in this manner, semi-infinite diffusion conditions are mimicked in the experimental setup. Upon formal analysis the concentration gradient developed was found to be linear dependent on the factor *It*^1/2^ (where I is the polarization current and t is the polarization time). It can be found upon the transformation of Equation (4) into a form denoted as Equation (5).

(4) c(x=0)=c∞+2t−0F(πD)12(Iti12)

(5) $$c\Delta c\;=\;c(x\;=\;0)\;-\;c_{\infty }$$

Moreover, the defined gradient Δ*c* regards each of the half cells (being in one case positive and negative in the other) to calculate the overall gradient observed in the cell (and therefore related to the potential difference observed for such an in situ created concentration cell) the Δ*c* value must be multiplied by two. Looking from the opposite side on Equation (6) one can easily notice that the anion transference number *t*^−^ can be determined for the *c*_∞_ if the size of the concentration difference is known. Unfortunately, the direct measurement of this value (similar to the one performed in the Hittorf method) is here impossible and an indirect approach based on the results of the previous concentration cells-based experiment must be applied.

Therefore, after a chosen time of polarization passed the current is interrupted and the potential value of the resulting cell is started to be measured as soon as it is possible. The value of interest is observed just after the discharge of the double layer capacitances but prior to the relaxation of the concentration gradients and the dependency of the ΔΦ on *It*^1/2^ is gathered. If the measurements are performed properly they should be linear in nature and characterized with the slope m. Finally, after incorporation of the previously defined slope parameter m the final dependency describing the anionic transference number is described by Equation (6):

(6) t−0 = mc∞F(πD)124(d cd lnU)

Finally, when the value of the transference number is known the mean molar activity coefficient of the salt can be extracted from the concentration cell data upon the application of Equation (7):

(7) $$\left(1+\frac{d\;lnf_{\pm }}{d\;lnc}\right)=\frac{-F}{2RTt_{-}^{0}}{\left(\frac{d\;U}{d\;lnc}\right)}_{}{}^{}$$

The method was pre-validated for the PEO-NaCF_3_SO_3_ system in 85 °C for the salt concentration range 0.14 to 2.58 mol/dm^3^ corresponding to the OE:Na ratios 160 to 8. The determined values of t_+_ were in range +0.31 (for the most diluted solution) down to −4.4 found for the most concentrated one. A local maximum was observed at *n* = 40 (0.56 mol/dm^3^) where the t_+_ value was equal to 0.09. Authors claim that results of this kind suggest the formation of the negatively charged mobile triplets consisting of two anions and one cation. In this situation, the mass transport of the sodium cation which occurs in the sample is directed in the same way as for all other negatively charged species, and therefore, against the natural direction of the mobility of the cation caused by the electric field gradient. However, considering the complexity of the results is unlikely that any simple speciation model would accurately describe this system. Upon a more detailed analysis of the results, the method is found to be valuable but very prone to experimental errors, especially those originating from the concentration cells’ potential measurements. Even a small experimental inaccuracy in these experiments results in the important deviations of the slope parameter (m), and thus, inflicts the values of the determined transference numbers.

Another sodium-based system was investigated by Ferry et al. in terms of basic measurements of the transport properties of the polymeric electrolytes [184]. The problem of the significance of the measurement uncertainties was further discussed by the same research group in its few next publications. A lithium triflate doped solid electrolytes based on the poly(propylene oxide) were studied [185] at 85 °C being in their quasi liquid form. A wide range of salt concentrations spanning from 0.06 to 5.65 mol/dm^3^ (corresponding to 400 to 2 PO:Li ratios) was investigated to discover the properties of systems being at different stages of predominant ionic associations. Unfortunately, it was found, that the experimental uncertainties of the determined transference numbers are large enough to make the result meaningless for all systems with PO:Li ratios higher than 15. Moreover, even below this threshold, these uncertainties were quite large reaching for a 1:3 sample situation in which the measured value is tenfold smaller than its uncertainty. Similarly, the values of the diffusion coefficients bear the deviations bars reaching more than half the order of magnitude. This situation makes, according to the opinion of the authors, the applicability of the method to this kind of system severely questionable.

Upon these quite non-promising conclusions, the need for deeper verification of the method validity arouses. Therefore, a model system—aqueous solution of silver nitrate was used to perform the reference experiments [181]. The system of this composition exhibits two main independent advantages: first, technical related to the well-defined composition of the electrolyte and stability of the potential of the silver electrode; second, more importantly, the fact that its thermodynamic factor can be independently derived from the salt activity coefficient value determined and not based on the above described electrochemical tests but from the measurements of the vapor pressure or the isopiestic measurements. Moreover, these data can be found in the literature. Therefore, the validity of the electrochemical determination of the most error-prone constituent of t_+_ value can be checked. A modified formula allowing us to calculate the transference numbers based on the independent thermodynamic data was here created and the results of both calculation schemes compared for two different solution concentrations being close to 0.1 and 1.0 M. It was found that the discrepancy between the results of both methods was equal to around 2% for the less concentrated solution and 4.5% in the case in the more concentrated one. On the other hand, the relative error of the fully electrochemically derived value was in both cases estimated to be twice as high (2%) in comparison with the externally supported one (around 1%). In addition to that, the statistical analysis of the internal integrity of the intermediate data was performed using the F statistical test. It was, as well, determined that the concentration-related dependencies of the diffusion coefficient assumed primarily to be linear are much better fitted with a more complicated polynomial equation. Therefore, upon the comparison of the results for these different systems, it must be finally concluded that the method itself is reliable but its reliability is strongly affected by the quality of the input experimental data which in the case of polymeric systems is questionably achievable.

Differently oriented verification was delivered in the contribution of Pesko et al. [186] originating from the same research team. The set of electrolytes utilizing PEO M_w_ = 5000 g/mol as a polymer host and LiTFSi as a dopant salt (EO:Li from 100 to 3.33) was investigated by means of three independent experimental techniques (PFG NMR, Bruce–Vincent and Newman’s) to compare the obtained values of the t_+_. It was found that both PFG NMR and B-V measurements yield positive and only slightly concentration-dependent (decreasing) values. On the other hand, Newman’s approach produces results of a totally different character. First of all, one can notice the negative values characterizing the samples with the highest salt concentrations, which (as was pointed out above) can be related to the predominant role of the negatively charged aggregates in this concentration range. The maximal positive values (being almost twice as high as the ones originating from the other two methods) being around 0.4 can be found in the middle concentration range while the most diluted samples exhibit lower but still positive values. For the most diluted solutions, they are even significantly lower than the reference results. In this case, the observed discrepancy is not explained by the authors. Moreover, it is worth stressing that in the whole concentration range the NMR originating values are significantly higher than the ones derived from the B-V polarization experiment. To understand this observation one should notice that these two methods were classified in [49] into two classes differencing in their sensitivity to the mobility of the neutral ionic species present in the sample.

#### 2.1.13. Modifications of Newman’s Approach

It is as well worth noticing that an interesting modification of Newman’s approach based on the utilization of a specially designed in-house manufactured four-electrode electrochemical cell is described in [161]. Two disc-shaped external electrodes serve here as the current terminals while two internal ones contacting the studied material through the internal surface of a very thin ring are used as voltage probes. This together with the specially designed polarization profile allows one to perform a complete set of measurements including conductivity, restricted diffusion, and potential of the in situ created concentration cell not only within one experimental setup but, as well, in the course of the one experiment. Consequently, the value of the t^+^ can be established based on one multi-stage experiment. On the other hand, it must be stressed that due to the nature of the system studied (not being a polymeric electrolyte, and therefore, being significantly less prone to the discrepancies related to the solution non-ideality) the transferability of the proposed methodology to the quasi-solid or highly viscous highly associated polymeric systems should be carefully validated.

In their another theoretical paper [187], the research group lead by Newman observed that while it is widely recognized by the scientific community that the complete characterization of concentrated, non-ideal (and, therefore, especially polymeric) electrolytes require properly set measurement of three independent transport properties, this is seldom done in practice. Moreover, upon the literature research, they conclude that in many cases the characterization of the transport properties of the studied materials is limited only to the determination of the overall ionic conductivity. This situation is confirmed by our own literature studies in which the homo-cationic conductivity of the various polymeric electrolytes is declared either without detailed studies in early work publications. The publications where transference number is determined for homo-cationic conductivity polymer electrolytes are based on Bruce and Vincent’s polarization experiments. It has to be emphasized that the Bruce and Vincent method is dedicated to polymer electrolytes and is facile in terms of sample preparation what is important for non-trivial electrolyte synthesis. However, to the author’s knowledge, there is no publication with analysis of different methods application in this type of electrolyte. Upon this observation the same authors [187] derive a dimensionless parameter N_e_ (Equation (8)):

(8) Ne=aκRT(t−0)12F2Dcc0cT

where: a—is related to salt concentration by:$=\nu /{\left(\nu_{+}z_{+}\right)}^{2}$ *v*—number of ions

*κ*—electrolyte conductivity at an initial salt concentration
*D*—diffusion coefficient of electrolyte based on thermodynamic factor
$c_{t}$—total solution concentration
$c_{0}$—concentration of monomers making up of polymer chain
c—salt concentration

Which can be used for an additional description of the charge transport phenomena occurring in the system, i.e., it is declared that the value of that parameter for an ideal case being the single ion conductor is equal to zero. Moreover, of two systems of the same conductivity are considered the one characterized with the lower Ne value is more appropriate for the practical application in the galvanic cell. The complete characterization of the polymeric ionic conductor suggested here is based on the measurement of diffusion coefficient D on the basics of the restricted diffusion experiment analogical to the one described earlier as a part of the determination of t_Ne_ and the Ne parameter based on the polarization experiment similar to the one proposed by Bruce and Vincent which results are corrected with the factor related to the activity coefficient of the salt in the electrolyte. The latter can be obviously determined using the experiment described earlier as another part of Newman’s method.

The practical application of the theoretical considerations mentioned above is delivered by Shah et al. in [188]. The approximate (Bruce and Vincent) and rigorously measured (which means the application of the corrections mentioned above) transference numbers were determined here for the fluorinated polymeric systems based on the (CF_2_CF_2_O)_n_ derived matrix and the LiFSI salt. Four independent measurements: conductivity, ideal transference number, concentration cell, and restricted diffusion were performed together and combined according to Equation (9):

(9) $$t_{+}^{0}=1-\sqrt{\frac{\frac{F^{2}\phi_{c}D_{s}c}{\nu \kappa_{s}RT}\left(\frac{1}{t_{+id}}-1\right)}{1+\frac{d\;ln\gamma_{\pm }}{d\;lnm}}}$$

where *ν* is a stoichiometric factor equal to 2 for a monovalent salt to determine the value of the rigorously measured transference number for cations.

The determined values of the ideal t_+_ decreased monotonically from 0.98 to about 0.65 with the increase of the salt concentration from 0.05 to 2.36 mol/dm^3^. On the other hand, the corrected values behaved completely differently rising from the strongly negative values lower than –1 observed for the most diluted solutions up to a still negative but close to 0 maximum at around 1.2 mol/dm^3^ and decreasing once again to around –0.25 for the most concentrated solution. A similar character comparison is delivered by Pesko et al. in their report [189] covering the studies on the PEO (M_w_ = 5000 g/mol and 275,000 g/mol) LiTFSI electrolyte. In this case, the B-V values of t_+_ were close to 0.17 for the most diluted solutions decreasing down to around 0.02 and increasing to about 0.25 for the most concentrated ones whole t_Ne_ (measured using two different approaches one described in [180] and the other in [186,187]) was in range 0 to 0.8 for diluted and concentrated solutions (where t_B-V_ was relatively high) and negative down to about –1.0 in the same concentration range where the former approach returned its minimal values. It was found that the latter methodology is even more prone to experimental errors than the original Newman’s approach as its results are dependent on the nature of the lithium electrode-electrolyte interface observed as the variations in its resistivity (R_SEI_). It is worth stressing that despite its great prospective applicational capabilities the Newman’s method, due to its experimental complication, was applied only to a limited number of systems.

### 2.2. Experimental

The C6P (1,1,3,3,5,5-meso-hexaphenyl-2,2,4,4,6,6-meso-hexamethylcalix[6]pyrrole) anion receptor was in-house synthesized in two-step synthesis proposed by Eichen et al. [190] which was improved in a manner described in [52]. It was later carefully vacuum dried (*p* = 10^−5^–10^−6^ Tr) for at least 60 h at temperature gradually increasing from ambient to about 120 °C. Modified inorganic Al_2_O_3_ based fillers with grafted acidic and basic surface groups (Sigma-Aldrich St. Louis, MI, USA, reagent grade) were pre-prepared according to [191] and dried in a vacuum oven (*p* = 10^−5^‒10^−6^ Tr, t = 200 °C). Polyether polymeric hosts PEGDME (poly(ethylene glycol) dimethylether) M_w_ = 500 g/mol and PEO (poly(ethylene oxide)) M_w_ = 4 × 106 g/mol Aldrich (St. Louis, MS United States, reagent grade) were dried similarly but with temperature not exceeding 60 °C. Salts such as LiBF_4_ LiI and LiClO_4_ (Aldrich St. Louis, MI, USA, reagent grade, 99.99%) were dried in a manner identical to the receptor. Acetonitrile (Aldrich St. Louis, MI, USA, battery grade, 99.93%, water content below 50 ppm) and dichloromethane (Aldrich St. Louis, MI, USA, biotech grade, 99.9%, water content below 20 ppm) were used as received. All the preparation steps were performed in an argon-controlled atmosphere dry-box having a humidity level below 5 ppm. PEO and salt were dissolved in acetonitrile while for C6P dichloromethane was used. Solutions were mixed together and solid polymeric membranes were prepared using a standard solvent casting technique and vacuum dried at 60 °C. Liquid samples were prepared by the dissolution of the appropriate amount of salt and C6P receptor in liquid PEGDME performed in 50 °C. In all cases, the amount of supramolecular additive (C6P) was determined based on the particular salt concentration to maintain the assumed anion to receptor ratio.

The conductivity of the solid and liquid samples was determined by means of the impedance spectroscopy performed in either stainless steel or titanium blocking electrodes from ambient to 120 °C and ambient to 70 °C temperature ranges respectively controlled by either a thermo-fan oven or HAAKE DC 50 cryostat respectively. PFG NMR experiments were performed as described in [52]. The transference numbers of the solid samples were determined using the Bruce–Vincent method with the details described in [52]. In all cases, the impedance spectra were analyzed using NLLS-based software to determine the material and process parameters. The details of such analysis are delivered in [192].

The transference numbers for the liquid samples were determined by both the Bruce–Vincent method and the Newman method. The B–V experiments were performed either in a classical manner when the impedance spectrum of the measured sample is registered only prior to and after the polarization period or applying an in-house developed measurement protocol within which the polarization current was delivered in half-hour periods between which an EIS experiment was multi times performed. In both cases, 20 mV polarization potential was applied whereas the intermittent impedance measurement-based approach was originally reported by us in [193]. On the other hand, the details of the application of Newman’s method are described in the original work of Ma (and Newman) et al. [180] and in our earlier work [194].

Technical details differed as we dealt with liquid, not semi-solid samples. Consequently, to facilitate concentration cell experiments, we designed a special cell. That consisted of two half cells made of polyethylene. The lithium electrode was placed in a special polypropylene “holder”. Electric contact is provided through a grid made of nickel wires. After completion of the electrode assembly, the electrolyte is put on each half cell which thereafter is merged and the OCV is measured. For the restricted diffusion and polarization experiments, a pair of identical electrode holders were separated with the silicon rubber o-ring type seal with a portion of the studied electrolyte in-between. The OCV values were registered either by VMP 3 or EG&G PAR 263 potentiostat devicesboth working in an OCV mode or using an in-house made pre-amplifier combined with a Brymen 515X registering microvoltmeter. That circuit delivered both the measured voltage amplification (with the amplification factor equal to 20) and the input impedance ranging around 1 TΩ. This allowed for the negligibly low load of the measured cells. These setups are later addressed as V, E, and P, respectively.

## 3. Results and discussion

The improvement of the cationic transport in polymeric electrolytes can be achieved using numerous methods including the addition of the ceramic fillers and supramolecular anion receptors (traps). In both cases, the addition of an additional component to the studied system makes it not only more complicated, but as well, more fragile to various discrepancies originating from the experimental factors of the electrochemical measurements. Therefore, it is valuable to gather results of the measurements of the cationic transference numbers performed by a set of methods (PFG, Bruce–Vincent’s, and Newman’s) to cross-verify their applicability to these systems. The importance of the comparison of this kind was mentioned above while addressing the report of Shigenobu et al. [98]. Finally, it must be noticed that these two particular electrochemical methods were chosen to be compared as the B–V approach even though it suffers from limitations related to the inadequacy of results in presence of mobile ionic pairs and charged triplets are extremely wide applied to such systems while the Newman’s approach should be, according to the claims of its authors, able to overcome the said drawbacks of the B–V technique.

At first glance, one should notice that the well-known discrepancy between t_+_ values originating from NMR and electrochemical measurements (ascribed, as it was stated above, to their different sensitivity towards mobile neutral species) become even more pronounced in the case of the samples containing the supramolecular additive. To highlight this deviation one should compare results gathered in Table 1 and Table 2 observing bolded corresponding values.

While the values characterizing the pristine system are quite similar (where the difference of 0.04 can be on the one appointed to the experimental error, and in addition, is in terms of its sign attributable to that inherent differences in sensitivities of both methods) the situation revealed upon the addition of the anion trap is noticeably various. Even though both methods reveal the expected improvement of the cationic transport two important issues should be considered. First is related to the magnitude of the observed difference being in this case about twofold. Secondly, the electrochemically determined value is higher than the spectroscopy originating one what should not take place if the difference mentioned above related to the sensitivity of the methods is considered. Therefore, to understand this one should notice the following observations. First of all the effect of C6P on the polymer host seems to be a mild plasticizing one—please compare the diffusion coefficients of the polymer host molecules notice the increase in the chain mobility. Moreover, one should take into consideration the ionic and complex formation equilibria present in the system of interest. Typical values for PEO-based systems [112] are equal to: K_I_ = 10^5^‒10^4^ kg*mol^−1^—ion pair formation constant; K_T_ = 10^1^–10^2^ kg/mol—ionic triplet formation; K_cal_ = 10^2^–10^3^ kg/mol—calix-anion complex formation constant. Thus, in the situation described with a series: K_I_ > Kcal > K_T_ only free and belonging to ionic triplets anions can form complexes. What at least in the latter case results in breaking of the transient crosslinks between macromolecules, and therefore, in the re-mobilization of the polymer chains. On the other hand, the ionic pairs are, at least directly, unaffected by the activity of the receptor. Consequently, the shift of the t_+_ value determined based on the mobility of the charged species (i.e., by the Bruce–Vincent’s polarization approach) is much more pronounced in comparison with the situation in which the neutral species (being unaffected by anion trapping) are counted as well. A more detailed description of how the presence of the anion receptor inflicts the PFG NMR image of the polymer electrolyte is delivered in [52]. A confirmation for this assumption can be found in a similar in nature analysis performed for the PEO-LiTf-C6P [159] system applying molecular spectroscopy experiments to determine the influence of the anion trap on the free ions—ionic pair—triplets equilibria. Parallelly the values of t_+_ were determined for the same systems using the Bruce–Vincent method. The transference numbers were here, as well, found to be significantly increased even by a small addition of C6P. In addition to that, the spectroscopic studies confirmed that the anion trap addition affects only the triplet-free ions equilibrium while the concentration of the ionic pairs remained almost intact.

In the next step, a set of t_+_ values determined by Newman’s method can be analyzed for systems incorporating both ceramic and organic modifiers. As it was previously mentioned the course of work, in this case, gathers three independent electrochemical experiments. The first of them was used to determine the diffusion coefficient of the salt using the polarization of the symmetrical cell using the direct current till a concentration cell is formed in situ through the motion of the ions in the electric field. After a certain time, the polarization was disengaged and the dependence (|ln(φ)| = f(t)) depicting the evolution of the potential during the “return to the equilibrium” state is recorded. The slope of the line is here directly proportional to the diffusion coefficient determined. To validate the accuracy of the method, three different experimental setups were involved to determine the magnitude of the discrepancies.

The results gathered in Table 3 are determined using the experimental approach proposed by Newman reveal the significant sensitivity of the determined value on the particular equipment setup used. It is worth considering that the values determined with the use of EG&G PAR 263 are about one magnitude higher than the other two result sets. This can be easily understood when one considers the influence of the input impedance, and thus, the current load of the concentration cell studied on the course of the measurement. In the ideal case: R = ∞ I = 0 the changes of the potential are related only to the diffusion originating equilibration of the just created concentration cell. If any finite current passes through the cell due to the load delivered by the measuring setup two additional phenomena occur. One of the most obvious ones is related to the potential decrease due to the over-potentials present in the system. The second and most important one is related to the fact that any current passing through the cell is related to the ionic transport occurring in the same direction as the diffusion originating one, and thus, accelerating the cell equilibration.

**Figure 1:**
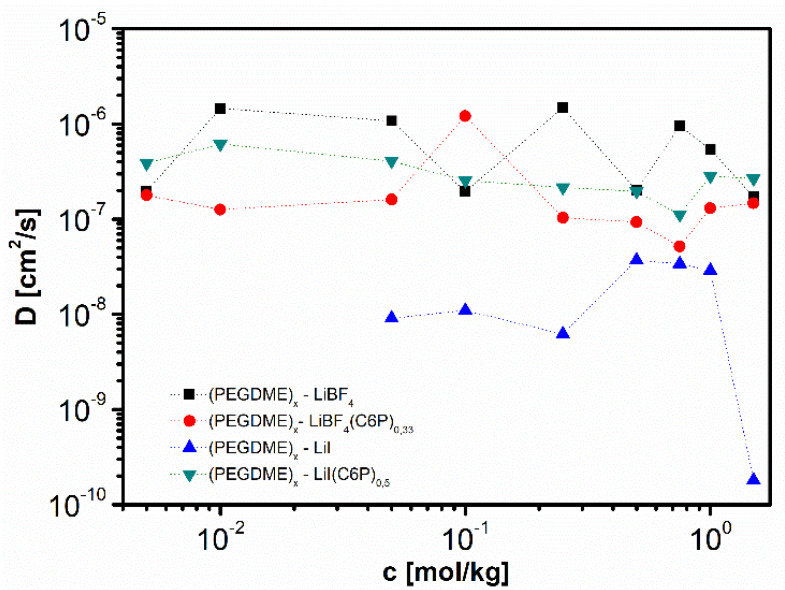
The values of the diffusion coefficients for poly(ethylene glycol) dimethylether (PEGDME)-LiX and PEGDME-LiX-C6P systems (LiI:C6P = 2:1, KiBF_4_:C6P = 3:1) as a function of the salt concentration for the anion trap based and reference electrolytes studied.

Finally, the set of the determined diffusion coefficient values for LiBF_4_ and LiI-based systems with and without the C6P addition is gathered in Figure 1.

It can be easily observed that the effect of the anion trap on the values of t_+_ is strongly dependent on the type of salt used. In the case of LiI, the addition of the anion trap promotes the diffusion of the salt in the electrolyte while in the case of the LiBF_4_ the effect is just the opposite. The observed discrepancy can be explained if one considers two possible mechanisms of the interaction of the trap with the electrolyte. Firstly, the direct one is related to the trapping, and thus partial immobilization of anions leading to the decrease of their diffusivity. The second, on the other hand, is related to the ionic triplets breaking, and through that, to the decrease of the number of the transient crosslinks between the polymer host molecules. This leads finally to the decrease of the viscosity of the system and, consequently, an iodide-containing system where the viscosity increases upon salt addition is much more prominent—the latter effect overrules the former while in the case of the tetrafluoroborate-based system the situation is just reverse. This explanation can be confirmed by the extremely low value of the diffusion coefficient observed for the most concentrated LiI-based electrolyte for which the transient crosslinks related increase of the viscosity is high enough to make its form gel-like. Moreover, upon the comparison of the results belonging to the series differencing in the type of the salt added it can be easily observed that for the LiI containing systems the addition of the anion trap (resulting in the transient crosslinks deterioration) leads to an increase of the salt diffusivity in a whole salt concentration range. Opposite to that in the case of the LiBF_4_ containing systems where the crosslinking is much less severe the changes in diffusivity are not only less prominent but, as well, their direction is opposite for most of the concentrations studied.

**Figure 2:**
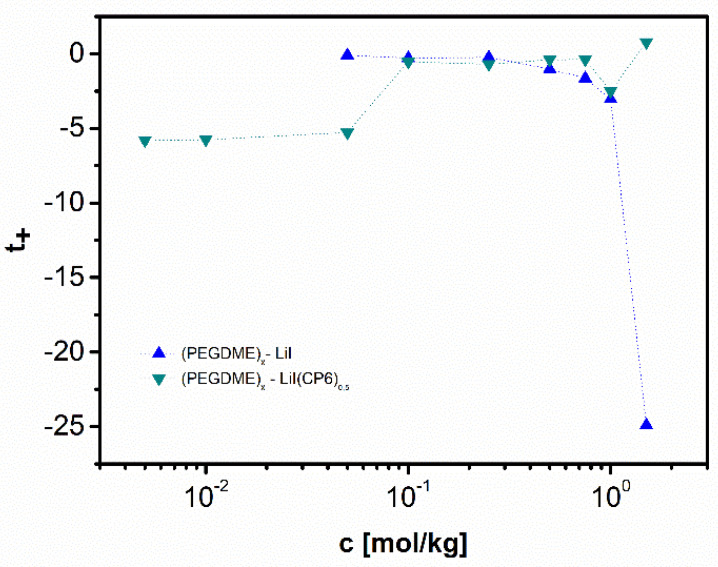
Cationic transference numbers determined using Newman’s approach as a function of the salt concentration for pristine and anion trap containing PEO DME LiI systems.

The results mentioned above together with two sets of concentration cell-based potential dependencies performed according to [195] allowed for the determination of the set of the transference numbers for all four systems studied. The first comparison can be delivered for the lithium iodide containing samples differencing with the addition of the anionic trap. In this case, the observed deviation (see Figure 2) in the cationic transport resulting from the anion immobilization are not so prominent. While the value obtained for the trap-free sample at 1.5 mol/kg is obviously outstanding from the whole set—which can be attributed to the viscosity increase related to severely lower diffusivity. On the other hand, despite the difference in the polymer matrix applied some similarities can be observed as the value obtained for this salt concentration for the anion trap containing sample (t_+_ = 0.77) is close to the ones determined for the solid PEO-LiI-C6P using the Bruce–Vincent method (t_+_ = 0.78 according to Table 1). The most significant change occurring upon the anion trap addition can be observed (see Table 4) in the middle range of the salt concentrations (0.5 to 1.0 mol/kg) where a slight but noticeable improvement of the cationic transport can be observed. It is worth noticing that for this highly associated salt this concentration range is the one where the ionic triplets play the predominant role in the overall charge carriers pool. It must be noticed, as well, that the values determined for the lowest salt concentration range as being much more prone to the experimental inaccuracies lead in the extreme cases to the impossibility of the determination of the diffusion coefficients which is the case for the two most diluted anion trap free samples.

**Figure 3:**
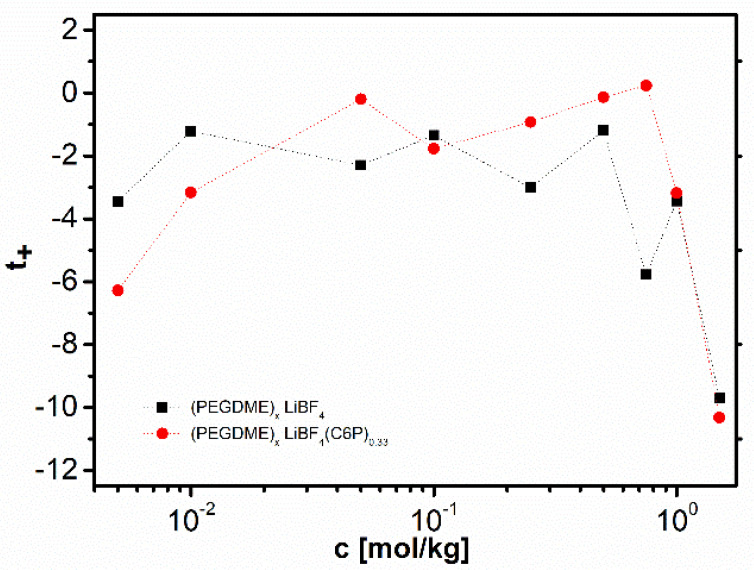
Cationic transference numbers are determined using Newman’s approach as a function of the salt concentration for pristine and anion trap containing PEGDME LiBF_4_ systems.

On the other hand, a similar set of experiments applied to the PEGDME-LiBF_4_ and PEGDME-LiBF_4_-C6P systems leads to results revealing the lithium transference numbers which are generally higher for samples containing the supramolecular additive (see Figure 3 and Table 5).

**Figure 4:**
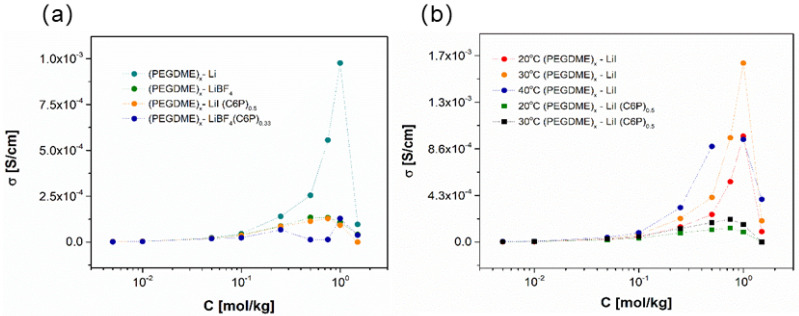
Values of the overall ionic conductivity as a function of the salt concentration for (**a**) pristine and anion trap containing PEGDME LiBF_4_ and PEGDME LiI systems determined at 20 °C (**b**) various temperatures for PEGDME LiI and PEGDME LiI(C6P)_0,5_ systems.

These (having in mind the difference mentioned above in the type of the applied matrix), when compared to the iodides-based systems, remain in much better correspondence with the results for the PEO-LiBF_4_-C6P solid system (see Table 1). This observation is especially true for samples with the highest conductivities (c_salt_ = 0.8–1.2 mol/kg—please refer to Figure 4 for details).

The determined improvement in the cationic transport is here (compare Figure 2 and Figure 3 or Table 4 and Table 5) much more pronounced than it was in the case of the LiI-containing samples. It is worth noticing that the observed discrepancy can be easily attributed to the difference in the complexation strength of the C6P molecules towards different anions being according to the quantum mechanics calculations and spectroscopic measurements presented in [166] much weaker in the case of iodides than in the case of tetrafluoroborate. On the other hand, the results reveal quite significant fluctuations in the t_+_ values determined for the anion trap-free samples characterized with the medium salt concentrations. This quite high sensibility to the experimental errors seems to be similar to the one reported by Newman et al. in [185]. It seems that the main problem observed here is related to the instability of the passivation layer on the lithium–electrolyte interface in the presence of even a small electric field. The growth of the passivation layer and change in overall resistance of sample related to this effect is not the only phenomenon we face. The formation of the passivation layer is partially an electrochemical reaction and, thus, decreases the current efficiency of the main process (lithium transport) is affecting, in consequence, the results obtained. Additionally, the charge carrier transport mechanisms within the SEI layer are different in comparison with the bulk electrolyte. Finally, we can assume that the measured value is an average of the properties of the electrolyte and of the layer which contributions to the overall value change with the growth of the passivation layer. It is worth stressing that the addition of the supramolecular compound is according to the research conducted previously (i.e., reported in [192]) an important factor leading to the stabilization of the SEI formation. Therefore, it is understandable that those deviations are significantly less pronounced for the anion trap containing series of samples.

Moreover, it should be noticed that in both presented cases the negative values of the determined cationic transference numbers can be explained by the assumption of a significant contribution of charged aggregates e.g., LiI_2_—triplets to the ionic transport. In the case of the charged species of this kind, the mass transport of lithium is coupled with the electric transport of the negative charge, and therefore, occurs in the reversed direction than in the case of the positively charged cations. Thus, the contribution of the negatively charged triplet to the lithium transference number is equal to −1. Taking into consideration that (i) multiple types of charge carriers are simultaneously present in the system, (ii) each charge carrier is characterized by its characteristic mobility, (iii) the concentration of these charge carriers is dependent on the concentration of salt and finally that (iv) lithium transference number should be calculated as the weighted average of all system constituents containing lithium one can expect that at least in some salt concentration ranges the obtained value will be negative or even lower than −1.

**Figure 5:**
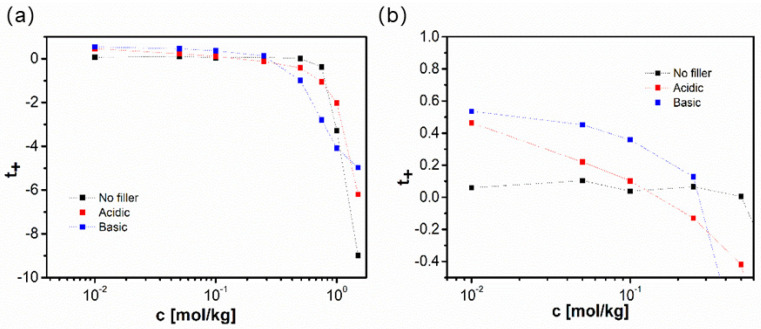
Cationic transference numbers determined using Newman’s approach as a function of the salt concentration for PEGDME LiClO_4_ modified Al_2_O_3_ systems (**a**). Magnification of the low salt concentration range (**b**).

The next investigated system comprised of PEGDME as a polymer matrix, LiClO_4_, and either none or one of two modified Al_2_O_3_ fillers (acidic and basic surface groups grafted) [194]. It is worth noticing that the choice of the different salt for this set of experiments is based on the differences in the strength of the anion-filler/receptor interactions for particular salts. While perchlorates are unable to be complexed by the C6P receptor neither iodides nor tetrafluoroborates exhibit significant affinity to the surface of the ceramic filler grains. The determined values of t_+_ for three sets of electrolytes are gathered in Figure 5. Based on the results presented here and the previous studies devoted to the same three constituent systems described in [183] it can be clearly seen that the drop in lithium transference number is observed in the salt concentration range in which the viscosity of the electrolyte studied has the dominant impact on the ion transport. This kind of behavior can be attributed to the interactions occurring between filler, salt originating species, and the polymer.

The said interactions lead to the diminishing of the strength and number of inter- or intramolecular crosslinks formed within the polymeric backbone by the salt originating positively charged triplets. Upon the presence of the filler, a disintegration of a positive triplet from the crosslink occurs leading, in consequence, to the release of two mobile cations, and therefore, to an increase in the lithium transference number. Moreover, considering that the surface groups of the filler can as well, act as the ion trapping receptors (basic sites can interact with cations while the acidic ones with anions), the modified filler is, at least hypothetically, able to break ion–ion connections in both triplets and ionic pairs. To understand the nature of these interactions one should consider the conductivity studies of the systems of interest reported previously by a group of Wieczorek [196,197,198]. From these investigations, it is evident that the increase in conductivity is related only to the salt concentration region in which a high degree of ionic associations (leading to the formation of charged triplets) is expected. Therefore, it can be assumed that the increase in conductivity results not from simple direct interactions of the filler with the ionic pairs (or free ions) but rather from more complicated in nature changes in ionic association due to ion-ion and ion-polymer interaction involving the inorganic filler. These interactions led not only to the lowering of the electrolyte viscosity but as well, to changes in the fraction of ionic associates present in the system and are ruled by two different mechanisms described in [199] occurring respectively for both types of the surfatypes of the surface groups grafted on the filler grains. Moreover, to understand the cationic transport enhancement caused by the addition of the basic filler to the samples of the lowest salt concentration range (domination of “free” ions in the overall charge carriers pole) one should notice that most of the cations in such a system are involved in the formation of the polymer-polymer crosslinks, and therefore, remain almost immobile. If a basic type filler is added these crosslinks are at least partially diminished and cations are bounded to the surface of the filler grains where their immobilization, hence present, is much weaker than in the pristine system. Therefore, in consequence, the addition of the cation binding additive is surprisingly increasing not decreasing their mobility and, as well, slightly increases the cationic transference number.

Moreover, the ionic association equilibrium determined for the systems of interest using the application of the Fuoss–Kraus formalism to the conductivity data (for details of which please refer to [199]) the activity of filler against the ionic pairs is significantly less probable in comparison with the one targeting the ionic triplets. Therefore, the positive effect of the addition of filler type additives can be observed in a high salt concentration range—1.5 mol/kg (being characterized with the predominant role of the ionic aggregates) and for the lowest salt concentrations where it acts as a free ion immobilization center. It is especially evident for composite electrolytes with fillers bearing acidic surface groups which can act as anion receptors (immobilizers). On the other hand, none or even reverse effect is observed in the intermediate salt concentration range in which ion-pairs are major constituents of the dissolved salt present in the system.

To conclude this set of observations one should notice that independently of their undoubtful importance results yielding from Newman’s approach are often affected by experimental errors of a significant magnitude. Independently on the particular system studied most of the results for lithium conductive systems suffer, therefore, from their limited reproducibility and reliability. Similar observations are reported in the previously cited works of Newman’s group [185,186]. Both in the case of the cited papers and our own research, most of the discrepancies observed, can be attributed to the measurements of the potentials of the concentration cells. The problem can be observed with a high dose of certainty related to the dependency on the salt concentration. It is revealed by the properties of the interphase (SEI) formed on the surface of the metal lithium electrode while in contact with the electrolyte components being thermodynamically unstable against it. The initial description of the phenomenon is backing to the originals contribution of Peled [200]. It was later explored by the same author [46], as well as, many other authors such as Yu et al. [201] and Nie et al. [202]. The details of the research conducted to reveal these dependencies in composite polymeric systems were, on the other hand, reported for example, in our previous papers [191,203].

Therefore it was worth investigating the systems of interest in terms of the deviations of the development of the passivation layer in time was analyzed for a PEGDME–LiI (0.75 mol/kg^3^) system. In this case, the salt concentration was appointed to the value at which the most practically significant effects of the addition of the supramolecular additive can be observed and the conductivity reveals the highest value (compare Figure 4). Three independent identical experiments were performed to determine the reproducibility of the process of interest.

**Figure 6:**
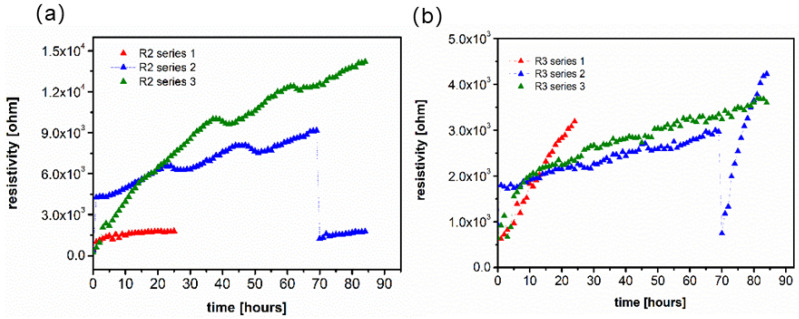
The time evolution of the resistivity of the passivation layer—R2 (**a**) and the charge transfer resistivity—R3 (**b**) for three independent polarization experiments performed on PEGDME LiI (0.75 mol/kg^3^) liquid electrolyte.

Figure 6 gathers three different time dependencies of passivation layer resistance (a) and charge transfer resistance (b). One can easily observe that the monotonical growth of the layer (Figure 6a) can be disturbed either by a kind of an oscillation process or by an abrupt depassivation process. Moreover, even in the most promising case of the monotonic growth of the layer the resistivity value remains unstable even after almost 100 hours of the polarization. On the other hand, the disturbances occurring in the passivation process inflict, as well, the kinetics of the electrochemical reaction on the electrode surface what can be correlated with the changes of the corresponding charge transfer resistance (see Figure 6b).

On the other hand, one must consider the importance of the in-time development and instability of the passivation layer is important not only in terms of the polarization type experiments mentioned above but, as well, in the case of the virtually current-less experimental conditions occurring in the set of experiments proposed by Newman et al. The first and obvious possible alteration resulting from the formation of SEI on the lithium electrode is related to its resistive character, and therefore to, the ohmic drop happening through it leading to the shift of the final value of the measured cell potential. One must, anyhow, notice that taking into consideration the values of the SEI resistance (not exceeding 10^4^) compared to the input resistance of the applied potential monitoring devices this kind of “kinetic” impact is truly negligible. On the other hand, the presence of the layer on the surface of the lithium electrode is, as well, important from the thermodynamic point of view. Both the thickness of the layer and its structure represent factors determining the final degree of hindrances in the lithium transport occurring through the SEI, and therefore, the value of the thermodynamic activity of the lithium metal “shielded” by the layer. This parameter is one hand directly undeterminable but on the other obviously correlated with the value of the SEI resistivity. Combining this observation with the mentioned above divergences of the R2 value for various salt concentrations one can easily conclude that the potential of the lithium electrode is dependent no only on the activity of lithium cations in the solution but, as well, on the activity of the lithium metal itself. Moreover, the latter factor is not only lower than the unitary value assumed by electrochemistry for solid pure metal electrodes but is varying with the changes in the concentration of the dissolved salt. Therefore, the thermodynamic potential of the electrode determined by Nernst’s equation is shifted not only with the changes of the activity of the lithium ions (which value is included in the numerator of the Nernst’s formula) but as well with the deviations of the activity of the metal, and therefore, by the non-unitary value of the denominator of the appropriate fractional expression denoted in the unabridged form of the equation. Therefore, one should additionally consider that the growth, as well as, the stabilization of the passivation layer is strongly influenced not only by the concentration of the salt [203] but also, by the addition of the inorganic fillers [184] or supramolecular anion traps [188]. It is, thus, easy to understand that significant and error-fertile discrepancies in the determination of the potential of the concentration cells for various juxtaposed combinations of electrolyte compositions can occur even in one experimental series. Moreover, this stipulation is consistent with the previously noticed observation delivered by Newman and coworkers attributing the highest error vulnerability to this part of the experimental setup. This observation is even more important upon a comparison of a different series of samples usually performed between a reference (additive-free) set and a one containing the additive of interest. Moreover, if one compiles this observation with the fact that sodium electrodes interfaced with the solid PEO based polymeric electrolytes are significantly less prone to the SEI development than the lithium ones the fact that the original research of Newman performed on the sodium systems is distinctly less affected with this kind of uncertainties than both his subsequent contributions and the research presented herein is easy to understand.

**Figure 7:**
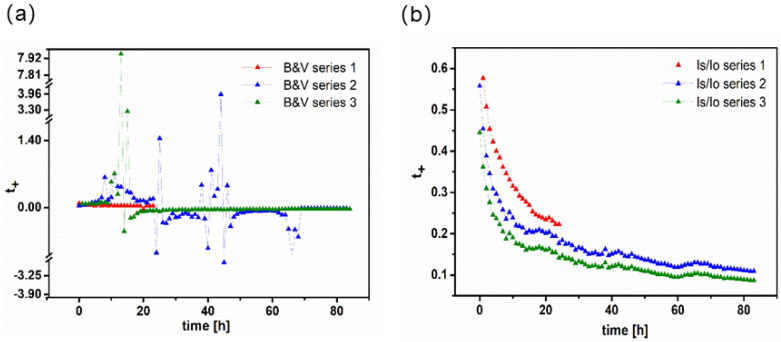
The time evolution of the values of the lithium transference numbers determined with a pristine polarization method (**a**) and Bruce–Vincent’s correction-based method (**b**) for three independent polarization experiments performed on PEGDME LiI (0.75 mol/kg^3^) liquid electrolyte.

The observations mentioned above are significant, as well, for the accuracy and reliability of the polarization type experiments performed according to the approach introduced by Bruce and Vincent. Therefore, it is worth verifying how these discrepancies occurring in the passivation of the lithium electrode reflect in the deviations of the values of the lithium transference numbers determined by both the plain polarization method and its Bruce–Vincent’s modification. For this purpose, a cyclically interrupted polarization experiment was performed. The electrolyte sample underwent a DC polarization of the same amplitude as in the standard polarization experiment in subsequent half-hour-long periods. During each interruption of the polarization, an impedance spectrum was gathered to both determine the above-discussed parameters and, as well, to gain the data for the B–V formula (see Equation (3)) based calculations of a “momentary” t_+_ value. Consequently, the value of t_+_ was calculated as a time-dependent value which in course of an undisturbed set of measurements should monotonically decrease down to a constant value. A comparison of the transference numbers determined based on the data registered within three independent experiments was gathered in Figure 7a. The values calculated based on the same experimental data omitting the correction proposed by Bruce and Vincent are, on the other hand, gathered in Figure 7b.

One can easily observe two important phenomena—one related to the fact that for the “pristine” manner of the t_+_ determination its value is not only significantly higher but as well, due to the persisting development of the passivation layer does not stabilize even at about near 100 hours of the experiment course.

**Figure 8:**
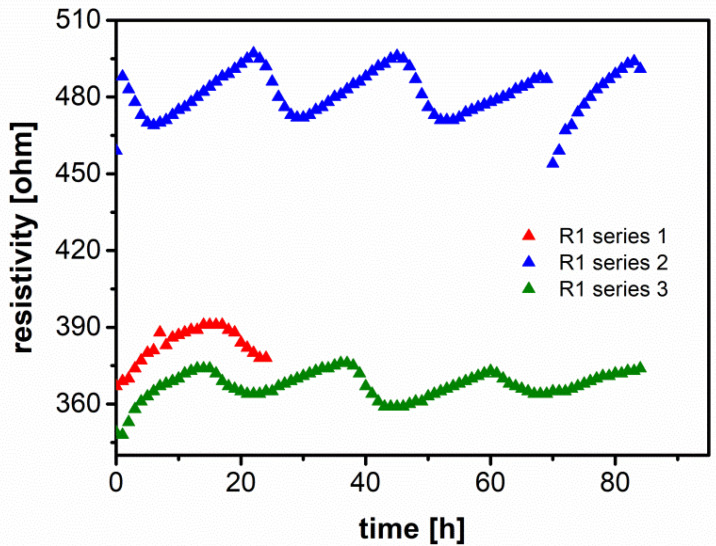
The time evolution of the resistivity of the electrolyte layer for three independent polarization experiments performed on PEGDME LiI (0.75 mol/kg^3^) liquid electrolyte.

The second important observation deals with the fact that the transference number values corrected by a Bruce–Vincent’s factor are not only significantly lower and much better stable at long polarization times but, on the other hand, are much more prone to the deviations related to the instability of the impedance parameters occurring in the course of the experiment. Moreover, another and even more important factor affecting the final results should be considered. It is related to the relative instability of the determined value of the electrolyte bulk resistivity (R1) (see Figure 8). Its impact seems to be even more important than the previously discussed behavior of the passivation layer. It is worth noticing that the experimental series yielding in the almost un-deviated values of t_+_ features the least abrupt changes of the R1 parameter with time. On the other hand, it is hard to unambiguously claim if the observed changes of the determined value of the electrolyte resistivity are related to the changes of the physicochemical properties of the sample or are rather a yield of the inaccuracies occurring during the application of the numerical procedures related to the spectra analysis.

## 4. Conclusions

At first, it is worth noting that unfortunately, the observed lithium transference number enhancement after the addition of the anion receptor in PEGDME-based systems is much smaller than in the case of solid PEO-based systems. It proves that independently of the similarities between solid and liquid systems the length of the polyether chain is important when the role of the anion receptor in ion transport properties is analyzed. In the case of the addition of the C6P receptor, an additional issue of system homogeneity must be taken into consideration due to the limited solubility of the receptor in liquid PEGDME. These observations are to some extent confirmed by the contribution originating from the same research group related to the anion trapping ability of the boron-based compounds [75]. Moreover, similar observations were made for many other composite systems e.g., those containing aluminum oxide-based fillers [160,191,199].

Looking more generally, one can find some observations being valid for all three sets of systems studied. Similarly, as in the case of pristine electrolytes, both anion traps, as well as, filler modified systems exhibit the t_+_ value decreasing with the increase of the salt concentration. Moreover, the determined t_+_ values are negative in the highest salt concentration ranges. As these negative values could be interpreted in terms of complexation of the lithium with the anionic species leading to the formation of negatively charged ion triplets it can be stipulated that the application of both types of ionic equilibria modifiers does not lead to the full deterioration of the ionic aggregates present in the studied electrolytes.

## Figures and Tables

**Table 1:** Bruce–Vincent’s lithium transference numbers of the P(EO)_N_(salt)_1_(C6P)_X_.

Salt		X	0	0.125	0.25	0.5	1.0
	N						
LiBF_4_	20		0.32	0.78	0.81	0.85	
	50				0.72	0.53	0.68
	100				1.06	0.75	0.92
LiI	20		0.24	0.56		0.78	
	50				0.75		
	100				0.49		

**Table 2:** Self-diffusion coefficients D for mobile species, polymeric host, and lithium transference number tNMRLi^+^. Salt to calixpyrrole ratio equal to 1:4.

	D Polymer 10^−8^ cm^2^/s	D− 10^−8^ cm^2^/s	D+ 10^−8^ cm^2^/s	t^+^
(PEO)_20_LiBF_4_-(C6P)_0,25_	6.51	27.5	24.6	**0.47**
(PEO)_20_LiBF_4_	3.37	36.1	20.0	**0.36**

**Table 3:** The values of the diffusion coefficient determined by various experimental equipment setups (V—VMP3 electrochemical analyzer, P—Brymen microvoltimeter/preamplifier, E—EG&G PAR 263).

Experiment	PEGDME LiI 0.1 mol/kg	PEGDME LiI 0.75 mol/kg
V1	3.35 × 10^−8^	1.97 × 10^−8^
V2	2.37 × 10^−8^	2.53 × 10^−8^
V3	4.35 × 10^−8^	2.44 × 10^−8^
V4	2.47 × 10^−8^	1.48 × 10^−8^
V5		2.46 × 10^−8^
E1	6.17 × 10^−7^	1.12 × 10^−7^
P1	2.25 × 10^−8^	1.18 × 10^−8^
P2	2.51 × 10^−8^	1.52 × 10^−8^

**Table 4:** Cationic transference numbers determined using the Newman approach as a function of the salt concentration for pristine and anion trap containing PEGDME LiI systems.

Concentration [mol/kg] (PEGDME)_x_-LiI	t_+_	Concentration [mol/kg] (PEGDME)_x_-LiI (CP6)_0,5_	t_+_
0.75	−1.65	0.75	−0.389
0.5	−1.02	0.5	−0.399
0.25	−0.25	0.25	−0.702
0.1	−0.29	0.1	−0.538
0.05	−0.1	0.05	−5.28

**Table 5:** Cationic transference numbers determined using Newman’s approach as a function of the salt concentration for pristine and anion trap containing PEGDME LiBF_4_ systems.

Concentration [mol/kg] (PEGDME)_x_-LiBF_4_	t_+_	Concentration [mol/kg] (PEGDME)_x_-LiBF_4_ (C6P)_0.33_	t_+_
0.75	−5.77264	0.75	0.23699
0.5	−1.19602	0.5	−0.13511
0.25	−3.00715	0.25	−0.92227
0.1	−1.34774	0.1	−1.77051
0.05	−2.29648	0.05	−0.20036
0.01	−1.21284	0.01	−3.16524
0.005	−3.45207	0.005	−6.28005

## Data Availability

Data available on request due to restrictions eg privacy or ethical. The data presented in this study are available on request from the corresponding author.
